# Characterization and treatment of polymicrobial biofilms in prosthetic joint infections

**DOI:** 10.1007/s00253-026-13985-w

**Published:** 2026-08-04

**Authors:** Estíbaliz Torrecilla–Sádaba, Andreia S. Azevedo, Daniel Carrizo, Isadora Maia, Laura Sánchez–García, Nuno F. Azevedo, Jaime Esteban, Llanos Salar–Vidal, John Jairo Aguilera–Correa

**Affiliations:** 1 Clinical Microbiology Department, Instituto de Investigación Sanitaria Fundación Jiménez Díaz, Autonomous University of Madrid (IIS-FJD, UAM), Avenida de los Reyes Católicos, 2, 28040 Madrid, Spain; 2 Laboratory for Process Engineering, Environment, Biotechnology and Energy (LEPABE), Faculty of Engineering, Associate Laboratory in Chemical Engineering (ALiCE), University of Porto, Rua Dr. Roberto Frias s/n, 4200–465 Porto, Portugal; 3 Centro de Astrobiología (CAB), CSIC–INTA, Calle Ajalvir, 28864 Torrejón de Ardoz, Madrid, Spain; 4 CIBERINFEC–CIBER de Enfermedades Infecciosas, Calle Monforte de Lemos, 3–5, Pabellón 11, Planta 0, 28029 Madrid, Spain; 5 Faculty of Biomedical and Health Sciences, Alfonso X El Sabio University (UAX), Avenida Universidad, 1, 28691 Villanueva de La Cañada, Madrid, Spain; 6 Department of Preventive Medicine, Faculty of Medicine, Public Health and Microbiology, Autonomous University of Madrid, Calle Arzobispo Morcillo, 4, 28029 Fuencarral–El Pardo, Madrid Spain

**Keywords:** Polymicrobial biofilms, Prosthetic joint infection, Biofilm modeling, PNA–FISH, Fatty acid analysis, Antibiotic combinations

## Abstract

**Abstract:**

Prosthetic joint infections are complex and associated with high economic and social costs. Polymicrobial cases remain poorly understood, particularly regarding bacterial interactions and antibiotic response within mixed-species biofilms. We introduce two approaches for characterizing polymicrobial biofilms: a predictive modeling equation and fatty acid profiling. Predictive modeling estimates biofilm behavior. Fatty acid profiling provided complementary information on species dominance and possible interspecies interactions. We integrate these results with fluorescence in situ hybridization and confocal microscopy to obtain a comprehensive view of biofilm structure and composition. Using this multi-modal approach, we characterize mono-, dual-, and triple-species biofilms formed by *Klebsiella pneumoniae*, *Pseudomonas aeruginosa*, and *Staphylococcus aureus*. In parallel, we evaluated common anti-biofilm antibiotics, alone and in combination, across all biofilm types. The model explained 72.9% of the variance in triple-species biofilm formation (*R*^2^ = 0.73).* K. pneumoniae* dominated all mixed biofilms in which it was present, accounting for approximately 70–80% of the bacterial population. In *P. aeruginosa*–*S. aureus* biofilms, *S. aureus* outnumbered *P. aeruginosa* (93% vs. 7%). Antibiotic efficacy varied with biofilm composition and structure. Colistin plus levofloxacin was the most effective combination, achieving reductions of up to 7.5 log_10_ CFU/cm^2^. *S. aureus* drove biofilm persistence; no treatment achieved eradication in mixed biofilms containing this species, with median reductions remaining below the 5 log_10_ CFU/cm^2^ threshold. Confocal microscopy revealed that *S. aureus* was predominantly located in the deepest biofilm layers. These results support two complementary approaches for characterizing polymicrobial biofilms through structural, predictive, and biochemical analyses and highlight the importance of biofilm architecture and interspecies interactions when evaluating antibiotic efficacy in polymicrobial infections.

**Key points:**

• *Modeling and fatty acid analysis predict polymicrobial biofilm behavior*

• *K. pneumoniae dominates biofilms, while S. aureus drives persistence*

• *Antibiotic efficacy depends on biofilm composition and structure*

**Graphical abstract:**

**Figure:**
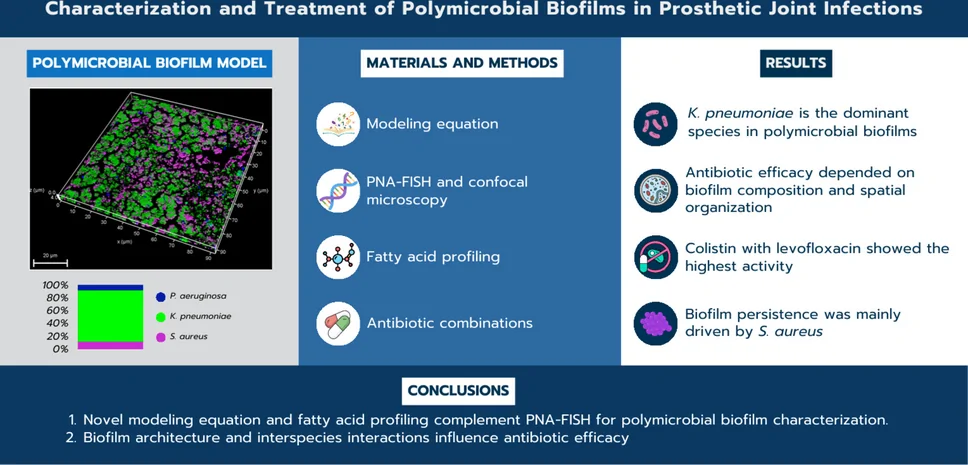

**Supplementary information:**

The online version contains supplementary material available at https://doi.org/10.1007/s00253-026-13985-w.

## Introduction

Prosthetic joint infections (PJIs) are serious complications after arthroplasty that cause substantial morbidity and societal costs (Hewlett et al. 2025). Among these, polymicrobial PJIs—defined as infections involving two or more microorganisms within the same joint—account for 6–37% of cases. The most frequent species in these infections are *Staphylococcus aureus*, coagulase-negative staphylococci (CoNS), *Enterococcus* spp., Gram-negative bacilli, and anaerobes (Flurin et al. 2019; Auñón et al. 2025).

Among implicated pathogens,* S. aureus* is frequently isolated in polymicrobial PJIs and shows higher recurrence rates than CoNS (Flurin et al. 2019; Hampton et al. 2024). Gram-negative bacilli are also more common in polymicrobial than in monomicrobial PJIs and are associated with worse outcomes (Marculescu and Cantey 2008; Auñón et al. 2025). Recently, an international Delphi consensus highlighted the clinical relevance of *Klebsiella pneumoniae*, *Pseudomonas aeruginosa*, and *S. aureus*, prioritizing them for research regardless of resistance (Cogliati Dezza et al. 2026). Therefore, these species were chosen for this study.

Despite their higher clinical complexity, polymicrobial PJIs remain poorly understood and underrepresented in guidelines. They also have lower cure rates than monomicrobial infections (Flurin et al. 2019; Auñón et al. 2025). As in monomicrobial PJIs, bacteria grow as biofilms—structured, surface-attached communities embedded in a self-produced matrix. In polymicrobial settings, interspecies interactions further enhance survival, reduce antibiotic susceptibility, and complicate eradication (Auñón et al. 2025).

Current therapeutic strategies mainly focus on antimicrobial selection, often overlooking how biofilm structure and ecology influence treatment response. Rifampicin and fluoroquinolones are the most studied agents in biofilm-related PJIs. Rifampicin primarily targets staphylococcal biofilms. Fluoroquinolones are commonly used against Gram-negative pathogens and serve as main partners for rifampicin in the treatment of staphylococcal PJIs. Colistin is relevant for Gram-negative biofilms, particularly those involving *P. aeruginosa* (Lora–Tamayo et al. 2019; Abad et al. 2020; Ferreira et al. 2024). However, the impact of biofilm organization and interspecies interactions on antibiotic efficacy in polymicrobial PJIs remains unclear.

To address these challenges, we use a clinically relevant three-species model—*K. pneumoniae*, *P. aeruginosa*, and *S. aureus*—to study polymicrobial biofilm behavior. We develop a predictive model of biofilm dynamics and use fatty acid profiling as a complementary approach to assess species dominance and explore potential interspecies interactions. We integrate these findings with peptide nucleic acid fluorescence in situ hybridization (PNA–FISH) and confocal microscopy. In parallel, we assess how biofilm composition, organization, and interspecies relationships affect the efficacy of anti-biofilm antibiotic therapies, both alone and combined.

## Materials and methods

### Bacterial strains and growth conditions

Collection strains were obtained from the American Type Culture Collection (ATCC, Manassas, VA, USA): *K. pneumoniae* ATCC 23357, *P. aeruginosa* ATCC 27853, and *S. aureus* ATCC 29213. All reference strains were stored in Difco™ Skim Milk (BD Diagnostic Systems, Sparks, MD, USA) at −20 °C. Frozen stocks were recovered by plating on tryptic soy agar (TSA) with 5% sheep blood (BioMérieux, Marcy l’Etoile, France). For use, isolates were grown in tryptic soy broth (TSB) (BioMérieux, Marcy l’Etoile, France) or on TSA with 5% sheep blood. Incubation was at 37 °C with 5% CO_2_ for 24 h.

The methodology described below complies with MIABiE recommendations (Lourenço et al. 2014).

### Biofilm formation

Biofilms were generated using a standardized protocol in all experiments. We included mono-, dual-, and triple-species combinations of *K. pneumoniae*, *P. aeruginosa*, and *S. aureus*. Each bacterial suspension was prepared in sterile 0.9% NaCl saline (Fresenius Kabi, Barcelona, Spain) at the concentrations listed in Online Resource Table S1. Suspensions were diluted 1:100 into brain-heart infusion (BHI) broth or cation-adjusted Müller–Hinton broth (CAMHB) for antimicrobial treatment experiments. Cultures were incubated under 5% CO_2_, in an anaerobic atmosphere for 24 h at 37 °C. This was done in a 2.5-L GENbox jar using CO_2_ atmosphere generator sachets (GENbox microaer, bioMérieux, Île-de-France, France).

Prior to biofilm formation, the medium and concentration were optimized (see Online Resource Fig. S1). This general workflow was adapted depending on the experimental purpose, as detailed in the corresponding sections below.

### Characterization of biofilms

#### Biomass quantification and predictive modeling equation

Biofilm formation was performed as described in the “Biofilm formation” section. Two hundred microliters of each bacterial mixture dilution in BHI was added to a sterile, untreated flat-bottomed 96-well plate (Falcon®, Thermo Fisher Scientific, Boston, MA, USA) and incubated for 24 h.

Biofilm biomass was quantified using a modified static biofilm formation assay as previously described (Stepanović et al. 2007). Briefly, wells were washed twice with 100 µL of sterile saline. Attached cells were fixed with 100 µL of methanol for 15 min at room temperature. Plates were then dried at 60 °C, stained with 50 µL of 2% (v/v) crystal violet for 10 min, and washed twice with 100 µL of sterile bidistilled water. The bound dye was eluted with 100 µL of absolute ethanol. Absorbance was measured at 570 nm using a TECAN Infinite 200 microtiter plate reader (Tecan Group Ltd., Männedorf, Switzerland). Two biological replicates and 24 technical replicates per biological replicate were performed. The experimental workflow is shown in Online Resource Fig. S2.

Biofilm formation was assessed using the optical density cutoff (ODc), calculated as previously described (Stepanović et al. 2007) (see Online Resource Fig. S3). Based on optical density (OD) values, strains were classified as non-, weak, moderate, or strong biofilm producers.

#### Spatial organization by PNA–FISH and confocal microscopy

To examine the spatial structure of biofilms, PNA–FISH was combined with confocal laser scanning microscopy. Three PNA probes, specific to each bacterial species’ rRNA sequences, were designed as previously described (Teixeira et al. 2021) (Table 1).

**Table 1:** PNA probes used in this study and their theoretical properties

Target	rRNA subunit	PNA probe sequence (5´–3´)	Melting temperature °C	Specificity (%)	Sensitivity (%)
*K. pneumoniae*	23S	Alexa Fluor 488–OO–CTACACACCAGCGT	75.79	98.93	91.76
*P. aeruginosa*	23S	ATTO 655–OO–CCTAGGTTATCCTAGG	74.19	99.63	98.13
*S. aureus*	16S	ATTO 550–OO–GGTGCACAGTTACTTAC	77.00	99.83	98.39

All probes were synthesized and HPLC-purified (> 90% purity, 25 nmol) by Eurogentec (Seraing, Belgium)

Probe specificity and channel separation were verified using a mixed-cell suspension containing all three bacterial species (See Online Resource Fig. S4). Biofilm formation was performed as described in the “Biofilm formation” section. Four hundred microliters of each bacterial dilution in BHI was transferred into an eight-well Lab-Tek II Chamber Slide w/Cover CC2 Glass Slide Sterile (Thermo Fisher Scientific, Rochester, NY, USA) and incubated for 24 h. After incubation, biofilms were gently rinsed once with saline to remove loosely attached cells. One biological replicate and one technical replicate per biological replicate were prepared.

The PNA–FISH procedure was performed according to a previously described protocol (Allkja and Azevedo 2021). Briefly, fixation and permeabilization were performed sequentially: thermal drying at 60 °C for 15 min, immersion in 100% methanol for 20 min, and fixation in 4% (v/v) paraformaldehyde at room temperature for 15 min. Samples were then incubated in 50% (v/v) ethanol for 15 min and air-dried at room temperature. For hybridization, biofilms were incubated in hybridization buffer. Each buffer contained 200 nM of the respective PNA probe. Incubation was performed in moist chambers at 60 °C for 1 h. Afterward, biofilms were washed in a pre-warmed wash buffer for 30 min at 60 °C. Once completely dry, a drop of immersion oil or mounting medium was applied onto the labelled biofilm. The sample was then covered with a coverslip and prepared for microscopic observation. Detailed buffer compositions are provided in Online Resource Table S2.

Samples were examined using a STELLARIS 5 confocal system mounted on a Leica DM6 B upright microscope (Leica Microsystems, Wetzlar, Germany) equipped with a tunable white light laser. Image acquisition was performed using a 63× oil-immersion objective. For each sample, at least five Z-stack images were acquired at randomly selected positions. Sequential scanning was used for mixed-species samples, and all acquisition settings were kept constant across experimental groups. Three-dimensional projections were generated using Leica LAS X software. Detailed acquisition parameters are provided in Online Resource Table S3. The experimental workflow is shown in Online Resource Fig. S5.

To compare the PNA–FISH experiment with colony-forming units (CFU) per area (CFU/cm^2^), the CFU/cm^2 ^values from the untreated positive control of the biofilm treatment experiment were used.

#### Fatty acid composition analysis

Biofilm formation was performed as described in the “Biofilm formation” section. Three mililiters of each bacterial dilution in BHI was transferred to a sterile, untreated six-well plate (Thermo Fisher Scientific, Rochester, NY, USA) containing 0.22-µm cellulose acetate membrane filters (47 mm in diameter; Sartorius, Göttingen, Germany), and the plate was incubated. Three biological replicates and one technical replicate per biological replicate were prepared.

Following incubation, the membranes were stored at − 20 °C until further processing. Fatty acid extraction of the frozen membranes was performed according to a previously described procedure (Grimalt et al. 1992; Sánchez-García et al. 2018; Sánchez–García et al. 2020). Two negative controls were included: membrane alone and membrane with BHI, to confirm the endogenous origin of the identified compounds.

Briefly, lipids were extracted from biofilm samples by sonication in an organic solvent mixture in the presence of an internal standard, followed by alkaline hydrolysis and subsequent acidification to recover the acidic fraction. This fraction was further extracted with an organic solvent, derivatized to fatty acid methyl esters, and analyzed by gas chromatography–mass spectrometry.

Molecular identification was performed using reference standards and spectral libraries, and quantification was carried out using external calibration curves. Recovery of the internal standards was 77 ± 10%. A schematic overview of the workflow and detailed extraction procedures and analytical conditions are provided in the Online Resource (Fig. S6, Table S4).

### Biofilm treatment

Biofilm formation was performed as described in the “Biofilm formation” section. Three mililiters of each bacterial dilution in CAMHB was transferred into sterile, untreated six-well plates and incubated for 24 h. After incubation, planktonic cells were removed by washing twice with sterile saline. Four biological replicates and one technical replicate per biological replicate were performed.

Biofilms were then treated with colistin, levofloxacin, and rifampicin, either individually or in all possible combinations. Treatments were applied at the highest reported plasma concentrations: colistin, 2.36 μg/mL (Garonzik et al. 2011); levofloxacin, 4 μg/mL (Chien et al. 1997); and rifampicin, 3.9 μg/mL (Dupouey et al. 2019). An untreated biofilm control was included in all experiments.

After 24 h of treatment, supernatants were collected to assess planktonic growth by measuring absorbance at 600 nm with a TECAN Infinite 200 microtiter plate reader. Wells were then washed twice with sterile saline solution to remove residual planktonic bacteria. Subsequently, biofilms were mechanically scraped. The scraping devices were then immersed in sterile saline solution and sonicated for 5 min to detach and disperse biofilm-associated cells. The resulting sonicated suspensions were used to determine bacterial CFU/cm^2^ using either the drop plate or the spread plate.

To enable selective recovery of each species, bacteria were plated on selective culture media: *K. pneumoniae* on MacConkey agar under anaerobic conditions, *P. aerugino*sa on MacConkey agar supplemented with 10 mg/L nalidixic acid, and *S. aureus* on mannitol salt agar (experimental workflow shown in Online Resource Fig. S7).

Bactericidal and eradication effects were defined as ≥ 3 log_10_ (Ishak et al. 2025) and ≥ 5 log_10_ (Konrat et al. 2016) reductions in CFU/cm^2^ compared with the control, respectively.

### Statistical analysis

Data analysis was performed using R (version 4.4.1) and/or GraphPad Prism (version 10.3.1). The normality of data was assessed using the Shapiro-Wilk test (for samples with fewer than 50 data points) or Kolmogorov-Smirnov test (for samples with more than 50 data points). Descriptive statistics are reported as the median and interquartile range for non-normally distributed data, and as the mean and standard deviation for normally distributed data.

For the biofilm quantification experiment described above, predictors for multi-species biofilm were selected via backward stepwise regression using the Akaike information criterion (Grane and Fortiana 2006). The predictors identified by the regression were then reintroduced into a linear model as independent variables, and the process was repeated until all of them were found to be significant (*P *< 0.05) (see Online Resource Fig. S8).

For multivariable comparisons, the nonparametric Dunn test with the Benjamini–Hochberg correction was used when the data did not follow a normal distribution. In contrast, the parametric one-way ANOVA was applied when the data followed a normal distribution. The significance level was set at 0.05.

## Results

### Characterization of biofilms

#### Biomass quantification and predictive modeling equation

**Fig. 1:**
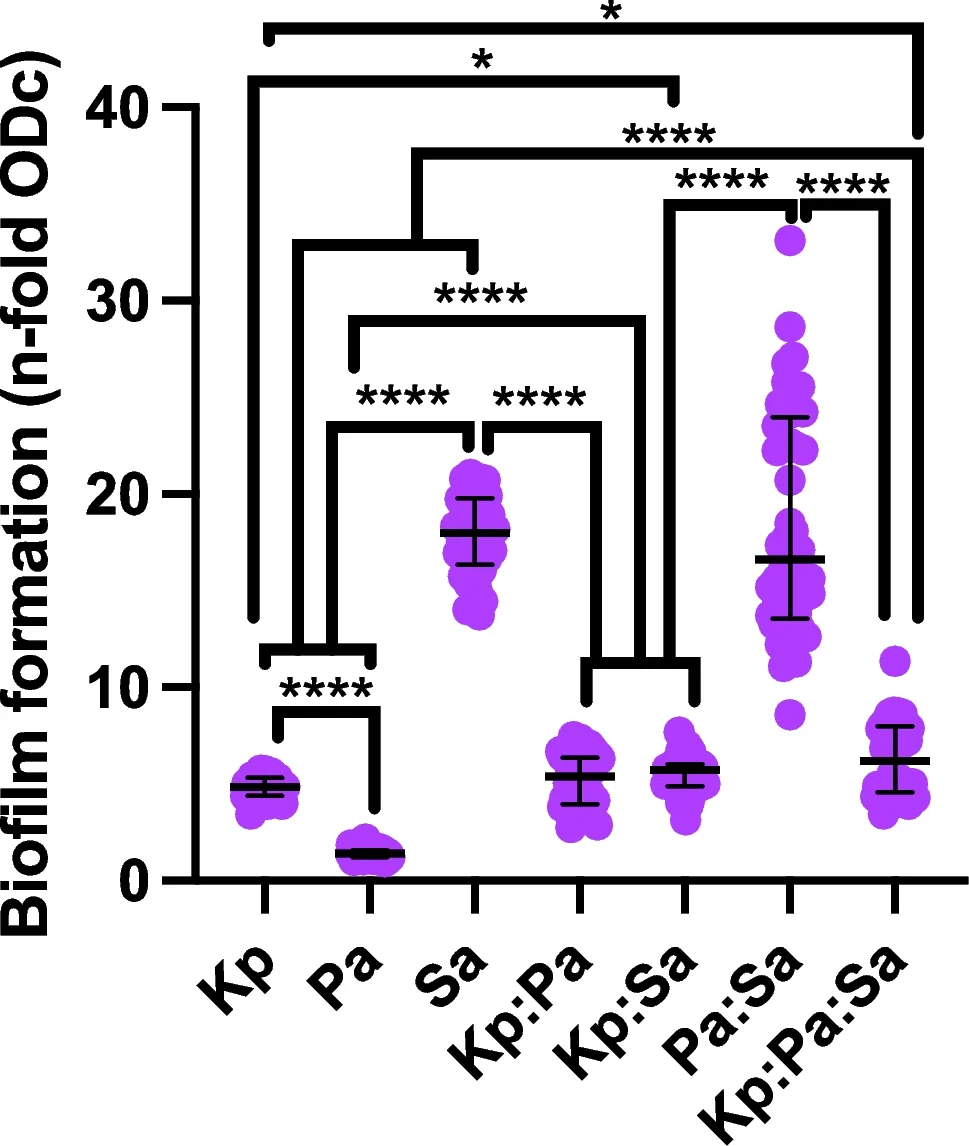
Quantification of biofilm formation in mono- and polymicrobial biofilms. ODc, optical density cut-off; Kp, *K. pneumoniae*; Pa, *P. aeruginosa*; Sa, *S. aureus*. Statistical significance is indicated as follows: *P < 0.05, **P < 0.01, ***P < 0.001, ****P < 0.0001. Data are presented as individual values with median and interquartile range

According to established classification criteria (Stepanović et al. 2007), *K. pneumoniae* ATCC 23357 was a strong biofilm producer (ODc 4.47), *P. aeruginosa* ATCC 27853 a weak producer (ODc 1.45), and *S. aureus* ATCC 29213 a strong producer (ODc 19.74). ODc distributions are shown in Fig. 1. Among the polymicrobial biofilms, the *P. aeruginosa*–*S. aureus* consortium showed the highest ODc values.

ODc parameters were used to select predictors for the triple-species biofilm through backward stepwise regression based on the Akaike information criterion (Grane and Fortiana 2006). The final regression model explained 72.9% of the variance (*R*^2^ = 0.73; *P *< 0.0001) and yielded the following equation:

$$\mathrm{K}\mathrm{p}:\mathrm{P}\mathrm{a}:\mathrm{S}\mathrm{a}\sim 0.792 \mathrm{K}\mathrm{p}:\mathrm{P}\mathrm{a}+0.79 \mathrm{K}\mathrm{p}:\mathrm{S}\mathrm{a}-0.199 \mathrm{S}\mathrm{a}$$

(Kp, *K. pneumoniae*; Pa, *P. aeruginosa*; Sa, *S. aureus*).

#### Spatial organization by PNA–FISH and confocal microscopy

**Fig. 2:**
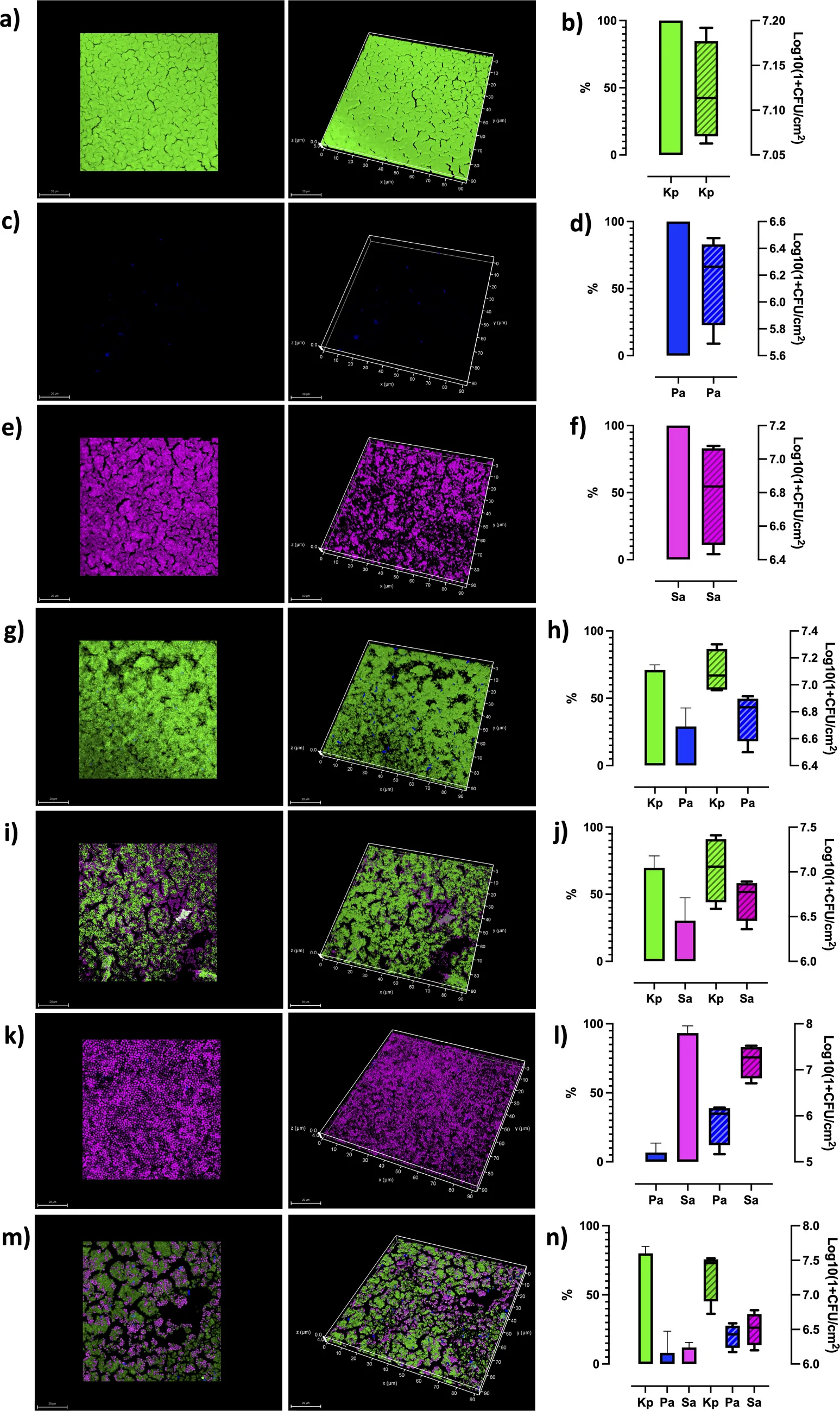
Confocal PNA–FISH analysis of mono- and polymicrobial biofilms. Confocal PNA–FISH images, percentage (solid bars) and bacterial load per area (CFU/cm^2^, striped box-and-whisker plots) of *K. pneumoniae* (Kp) biofilm (**a**, **b**), *P. aeruginosa* (Pa) biofilm (**c**, **d**), *S. aureus* (Sa) biofilm (**e**, **f**), dual-species biofilms (Kp:Pa, **g**, **h**; Kp:Sa, **i**, **j**; Pa:Sa, **k**, **l**), and triple-species biofilm (Kp:Pa:Sa, **m**, **n**). Solid bars represent the median and interquartile range. Striped box-and-whisker plots represent the median and interquartile range, with whiskers indicating the minimum and maximum values

Confocal PNA–FISH results for the relative abundance of biofilm, expressed as percentage and CFU per area (CFU/cm^2^), are shown in Fig. 2.

In mono-species biofilms, *K. pneumoniae* formed the densest layer (7.11 log_10_ CFU/cm^2^), followed by *S. aureus* (6.84 log_10_ CFU/cm^2^), whereas *P. aeruginosa* showed the lowest density (6.26 log_10_ CFU/cm^2^) (Fig. 2a–f).

*K. pneumoniae* predominated in both dual-species biofilms, accounting for 70.9% of the population in the *K. pneumoniae*–*P. aeruginosa* biofilm and 69.6% in the *K. pneumoniae*–*S. aureus* biofilm. In both cases, *K. pneumoniae* formed the densest layer, occupying the basal layer in the *K. pneumoniae*–*P. aeruginosa* biofilm, with *P. aeruginosa* forming surface clusters above, whereas in the *K. pneumoniae*–*S. aureus* biofilm, *K. pneumoniae* occupied the upper layer and *S. aureus* the deeper layers (Fig. 2g, h and Fig. 2i, j, respectively). In the *P. aeruginosa*–*S. aureus* biofilm, *S. aureus* predominated, accounting for 93.3% of the population and forming the bottom layer, whereas *P. aeruginosa* appeared as scattered aggregates above (Fig. 2k, l).

In the triple-species biofilm, *K. pneumoniae* was the most abundant species, accounting for 80% of the population, whereas *S. aureus* and *P. aeruginosa* were present at lower abundances (11.9% and 8.1%, respectively). The biofilm exhibited a layered architecture, with *S. aureus* at the bottom, *K. pneumoniae* in the middle, and *P. aeruginosa* at the surface (Fig. 2m, n).

#### Fatty acid composition analysis

**Fig. 3:**
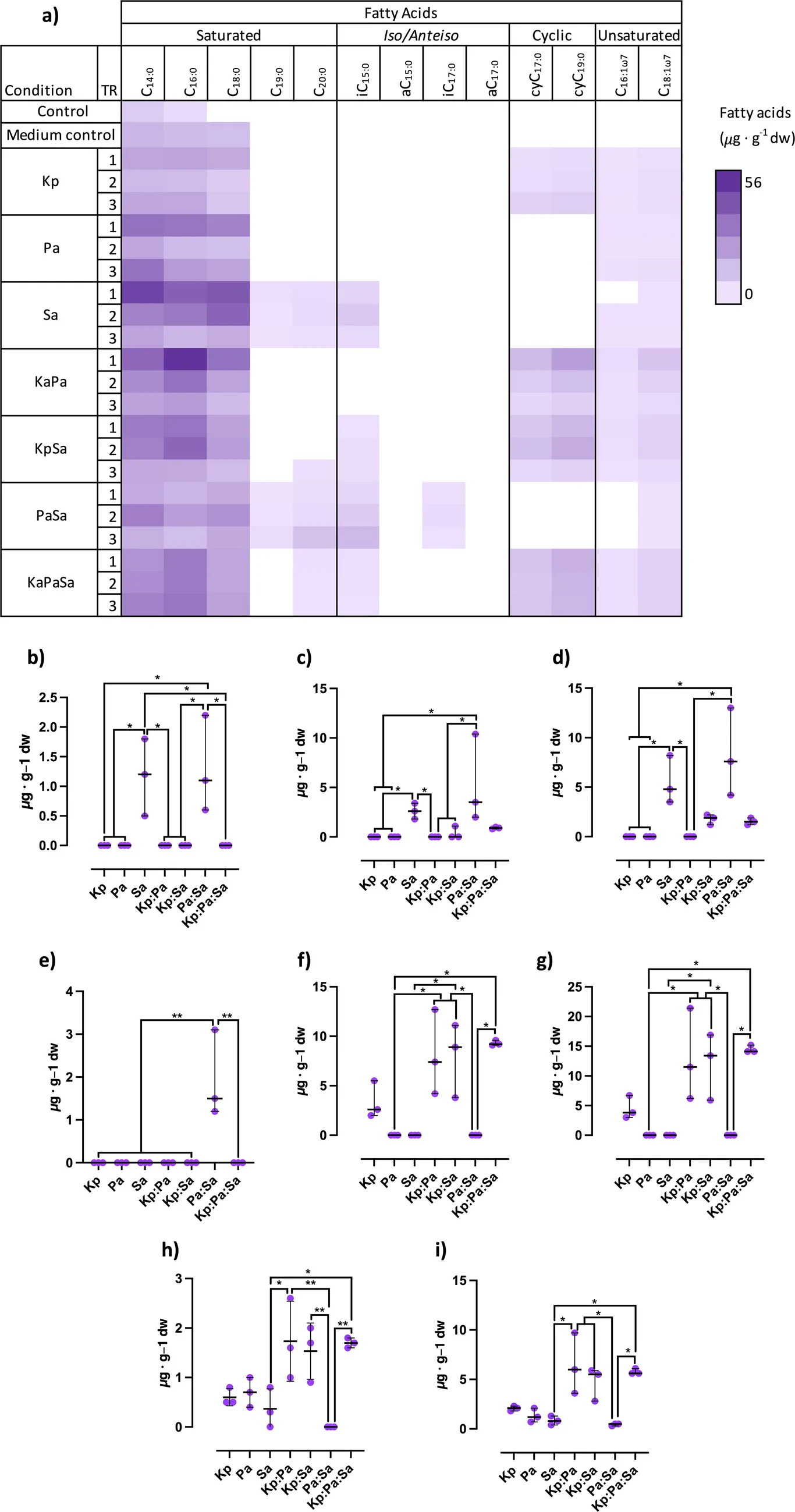
Fatty acid composition of mono- and polymicrobial biofilms. **a** Composition and concentration (μg·g^−1^ dry weight) of specific fatty acids in biofilms of *K. pneumoniae*, *P. aeruginosa*, *S. aureus*, and their combinations. **b** C_19:0_. **c** C_20:0_. **d** *i*C_15:0_. **e** *i*C_17:0_. **f** cyC_17:0_. **g** cyC_19:0_. **h** C_16:1ω7_. **i** C_18:1ω7_. Kp, *K. pneumoniae*; Pa, *P. aeruginosa*; Sa, *S. aureus*. Statistical significance is indicated as follows: *P < 0.05, **P < 0.01, ***P < 0.001, ****P < 0.0001. Data are presented as individual values with the median and interquartile range, except for C_16:1ω7_, which is presented as the mean and standard deviation

Fatty acid concentrations (μg·g^−1^ dry weight) are shown in Fig. 3.

Saturated fatty acids, C_19:0_ and C_20:0_, were detected exclusively in *S. aureus* mono-species biofilms. C_19:0_ and C_20:0_ reached concentrations of 1.2 and 2.6 μg·g^−1^ dry weight, respectively. Similar concentrations were observed in *P. aeruginosa*–*S. aureus* biofilms (1.1 and 3.5 μg·g^−1^ dry weight, respectively), whereas both fatty acids were detected at lower or negligible levels in *S. aureus* biofilms containing *K. pneumoniae* (Fig. 3b, c). The branched *iso*-fatty acid *i*C_15:0_ showed a similar pattern, with higher levels in *P. aeruginosa*–*S. aureus* biofilms than in *S. aureus* mono-species biofilms (7.6 vs. 4.8 μg/g^−1^ dry weight) (Fig. 3d). The branched *iso*–fatty acid *i*C_17:0_ was detected only in *P. aeruginosa–S. aureus* biofilms (Fig. 3e).

Cyclic fatty acids, cyC_17:0_ and cyC_19:0_, were exclusive to *K. pneumoniae* mono-species biofilms, reaching concentrations of 2.6 and 3.8 μg·g^−1^ dry weight, respectively. These fatty acids were more abundant in multi-species biofilms containing this species, reaching 7.4 and 11.5 μg·g^−1^ dry weight in *K. pneumoniae*–*P. aeruginosa* biofilms, 8.9 and 13.4 μg·g^−1^ dry weight in *K. pneumoniae*–*S. aureus* biofilms, and 9.2 and 14.1 μg·g^−1^ dry weight in triple-species biofilms (Fig. 3f, g).

Monounsaturated fatty acids, C_16:1ω7_ and C_18:1ω7_, were detected in all mono-species biofilms, with higher levels in Gram-negative species than in *S. aureus*. *K. pneumoniae* and *P. aeruginosa* reached concentrations of 0.6 and 2.1, and 0.7 and 1.2 μg·g^−1^ dry weight, respectively, whereas *S. aureus* showed lower values (0.4 and 0.8 μg·g^−1^ dry weight). In multi-species biofilms, both fatty acids increased in biofilms containing *K. pneumoniae*, reaching 1.7 and 6 μg·g^−1^ dry weight in *K. pneumoniae*–*P. aeruginosa* biofilms, 1.5 and 5.5 μg·g^−1^ dry weight in *K. pneumoniae*–*S. aureus* biofilms and 1.7 and 5.6 μg·g^−1^ dry weight in triple-species biofilms. In contrast, they were absent (C_16:1ω7_) or detected at very low levels (C_18:1ω7_) in *P. aeruginosa*–*S. aureus* biofilms (Fig. 3h, i).

### Biofilm treatments

**Fig. 4:**
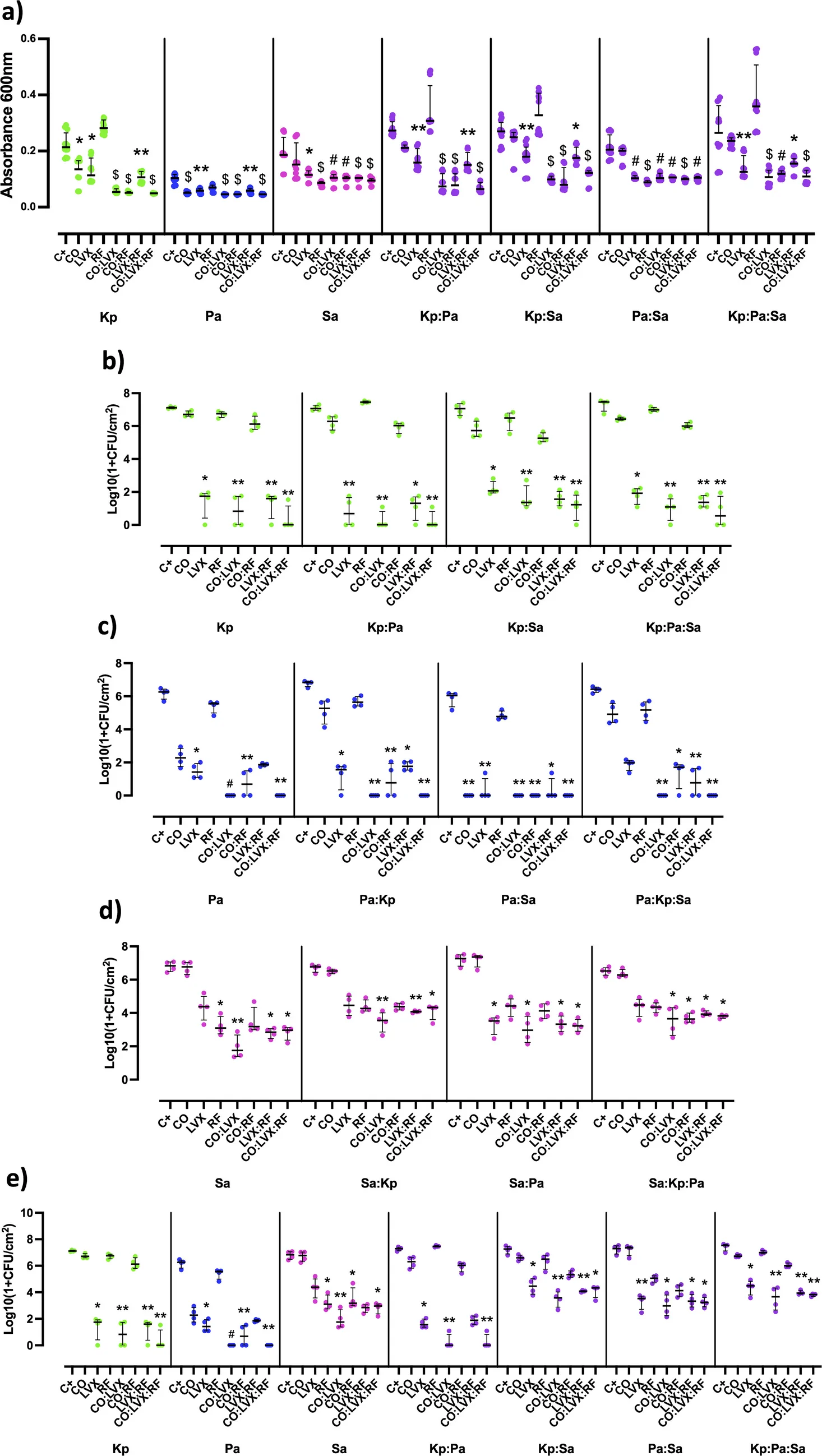
Antimicrobial effects on mono- and polymicrobial biofilms. CFU per area (CFU/cm^2^) in mono- and polymicrobial biofilms for each bacterial species. **a** Optical density at 600 nm of planktonic cells. **b** CFU/cm^2^ of *K. pneumoniae* (Kp). **c** CFU/cm^2^ of *P. aeruginosa* (Pa). **d** CFU/cm^2^ of *S. aureus* (Sa). **e** Total CFU/cm^2^ (sum of all species). C+, positive control; CO, colistin; LVX, levofloxacin; RF, rifampicin. Statistical significance is indicated as follows: *P < 0.05, **P < 0.01, #*P *< 0.001, $*P *< 0.0001. Data are presented as individual values with the median and interquartile range

Figure 4 summarizes the effects of the different antimicrobial regimens compared to the positive controls. Detailed statistical comparisons appear in Online Resource Fig. S9.

Under planktonic conditions, levofloxacin and all combinations inhibited all cultures. Colistin inhibited *K. pneumoniae* and *P. aeruginosa* mono-species cultures. Rifampicin inhibited *S. aureus* mono-species cultures and its dual-species culture with *P. aeruginosa* (Fig. 4a).

For *K. pneumoniae*, levofloxacin was bactericidal and eradicative in all biofilms (5.6 log_10_ CFU/cm^2^ reduction), except in dual-species biofilms with *S. aureus*, where it was only bactericidal (4.7 log_10_ CFU/cm^2^ reduction). All combinations except colistin-rifampicin were both bactericidal and eradicative, with reductions of 5.7–7.0 log_10_ CFU/cm^2^ (Fig. 4b).

For *P. aeruginosa*, levofloxacin and all combinations were bactericidal across biofilms, with reductions of 4.9–6.4 log_10_ CFU/cm^2^. Eradication occurred in all cases except mono-species biofilms treated with levofloxacin–rifampicin (4.4 log_10_ CFU/cm^2^ reduction). Colistin alone was bactericidal only in mono-species (4 log_10_ CFU/cm^2^ reduction) and *P. aeruginosa*–*S. aureus* dual-species biofilms, achieving eradication only in the latter (6.1 log_10_ CFU/cm^2^ reduction) (Fig. 4c).

For *S. aureus*, rifampicin and all combinations were bactericidal in mono-species biofilms, resulting in reductions of 3.5–5.1 log_10_ CFU/cm^2^. In dual-species with *K. pneumoniae*, only colistin–levofloxacin was bactericidal (3.3 log_10_ CFU/cm^2^ reduction). In dual-species with *P. aeruginosa*, levofloxacin and all combinations were bactericidal, resulting in reductions of 3.0–4.1 log_10_ CFU/cm^2^. No treatment was bactericidal in triple-species biofilms. Eradication did not occur under any condition, except with colistin–levofloxacin in mono-species biofilms (5.1 log_10_ CFU/cm^2^ reduction) (Fig. 4d).

Considering total bacterial counts in mixed biofilms, levofloxacin and all combinations except colistin–rifampicin were bactericidal, except in the *P. aeruginosa*–*S. aureus* dual-species biofilms (where colistin–rifampicin was bactericidal, 3.0 log_10_ CFU/cm^2^ reduction) and triple-species biofilms (where levofloxacin was not, 2.9 log_10_ CFU/cm^2^ reduction). Eradication occurred only in *K. pneumoniae*–*P. aeruginosa* biofilms with levofloxacin and all combinations except colistin–rifampicin, with reductions of 5.4–7.3 log_10_ CFU/cm^2^. No eradication was observed in mixed biofilms containing *S. aureus* (Fig. 4e).

## Discussion

In this study, we analyzed mono-, dual-, and triple-species biofilms formed by *K. pneumoniae*, *P. aeruginosa*, and *S. aureus*. The main findings indicate that *K. pneumoniae* appears to determine overall biofilm architecture, *S. aureus* drives antibiotic resistance in mixed communities, and spatial organization, together with interspecies interactions, shapes antimicrobial responses. These results, obtained through PNA–FISH, predictive modeling, and fatty acid profiling, provide a framework for understanding and predicting treatment outcomes in polymicrobial infections.

Microorganism interactions range from competition to cooperation (Liu et al. 2016). Using the predictive equation, we found that *K. pneumoniae* connects *P. aeruginosa* and *S. aureus* within the biofilm, acting as a structural nexus, while *S. aureus* functions as a biofilm disruptor. These findings are consistent with the spatial organization observed by PNA–FISH and supported by CFU quantification. The predictive equation, therefore, provides a novel framework for inferring interspecies relationships in polymicrobial biofilms.

PNA–FISH combined with confocal microscopy is a widely used approach for analyzing the three-dimensional structure of polymicrobial biofilms. Different spatial organizations have been described, including segregated microcolonies, mixed communities, and layered structures (Allkja and Azevedo 2021). In our model, spatial analysis revealed a layered biofilm structure, with *K. pneumoniae* consistently showing an ecological advantage in mixed biofilms. This is consistent with previous reports describing neutral interactions with *P. aeruginosa* and weak antagonism with *S. aureus* (Dos Santos Goncalves et al. 2014). In contrast, the *P. aeruginosa*–*S. aureus* interaction displayed a predominantly antagonistic pattern, with *S. aureus* emerging as the dominant species. Under anaerobic and surface-associated conditions, *P. aeruginosa* likely reduces growth and virulence, persisting as small aggregates rather than forming its typical mushroom-shaped architecture (Stacy et al. 2016; Pallett et al. 2019; Niggli et al. 2021), as observed in our model.

Consistent with PNA–FISH and the predictive model, fatty acid profiles provided complementary information on species dominance within biofilms. Fatty acids are key components of bacterial membranes involved in environmental adaptation and biofilm formation (Chao et al. 2010). Long saturated and branched fatty acids are characteristic of Gram-positive bacteria, whereas cyclic and monounsaturated fatty acids are typical of Gram-negative species (Gharaibeh and Voorhees 1996). The distribution of long saturated and branched fatty acids reflected species dominance, with *S. aureus* prevailing in the *P. aeruginosa*–*S. aureus* biofilm and *K. pneumoniae* dominating when present. Branched fatty acids increase membrane fluidity and are essential for *S. aureus* survival under stress and nutrient limitation, which may explain the higher proportion of *i*C_15:0_ and the presence of *i*C_17:0_ in the *P. aeruginosa*–*S. aureus* biofilm (Sen et al. 2016; Frank et al. 2021). In contrast, cyclic and monounsaturated fatty acids were more abundant in biofilms containing *K. pneumoniae*, supporting its dominant role in mixed communities. Cyclic fatty acids, derived from monounsaturated fatty acids, typically increase under stress or nutrient limitation (Chao et al. 2010). Although cyclic fatty acids were not detected in *P. aeruginosa* in our experiments, this species is known to synthesize them, albeit at lower levels than enterobacteria (Gharaibeh and Voorhees 1996). Therefore, the increased abundance of cyclic fatty acids in mixed biofilms containing *K. pneumoniae* may reflect enhanced synthesis by this species and/or contributions from *P. aeruginosa*. While *K. pneumoniae* and *P. aeruginosa* can synthesize monounsaturated fatty acids, *S. aureus* relies on external sources. Thus, the increase in mixed biofilms containing *K. pneumoniae* may result from enhanced production by Gram-negative bacteria under stress and/or the acquisition of exogenous monounsaturated fatty acids by *S. aureus* (Chao et al. 2010; Sen et al. 2016). The absence of cyclic and monounsaturated fatty acids in the *P. aeruginosa*–*S. aureus* biofilm may be related to the marked reduction in *P. aeruginosa* abundance observed in this dual-species biofilm compared with the corresponding mono-species biofilm. This reduction could be associated with competitive interactions with *S. aureus*, leading *P. aeruginosa* to enter a physiologically less active state in which fatty acid production decreases (Chao et al. 2010). Overall, fatty acid profiling complements spatial analysis by providing insight into species dominance and potential physiological adaptation in polymicrobial biofilms.

In terms of antimicrobial susceptibility, biofilm composition, spatial structure, and the dominant bacterial species strongly influenced treatment efficacy. Monotherapy was generally ineffective against mixed-species biofilms, whereas combination therapies showed improved activity; however, the presence of *S. aureus* reduced overall treatment efficacy. Most monotherapies that were active against planktonic cells lost their activity in the biofilms. Levofloxacin was the only antibiotic that retained substantial activity as monotherapy across most biofilm types, likely due to its broad antimicrobial spectrum and intracellular mechanism of action (Croom and Goa 2003). The loss of colistin activity in *K. pneumoniae* biofilms may be related to heteroresistance, i.e., the emergence of resistant subpopulations during biofilm formation under colistin exposure (Silva et al. 2016). Another possible explanation is that colistin requires metabolically active cells to exert its bactericidal effect and reach the inner membrane. Therefore, the reduced metabolic activity of bacteria located in the deeper layers of the biofilm may contribute to increased tolerance to colistin treatment (Borrelli et al. 2025). In contrast, in *P. aeruginosa*, decreased activity in mixed biofilms with *K. pneumoniae* may be associated with structural changes, including loss of the typical mushroom-like architecture and formation of flat aggregates, which are less susceptible to colistin (Armengol et al. 2023). Rifampicin showed reduced activity against *S. aureus* in mixed biofilms, likely due to limited penetration when other species occupy upper biofilm layers. However, in the *P. aeruginosa*–*S. aureus* biofilm, increased susceptibility to all antibiotics was observed, likely due to antagonistic interactions between both species (Trizna et al. 2020). Among combinations, colistin–levofloxacin was the most effective. In Gram-negative bacteria, colistin disrupts the outer membrane, and in *S. aureus*, colistin destabilizes the biofilm matrix, allowing levofloxacin to penetrate (Klinger–Strobel et al. 2017; Armengol et al. 2023). This colistin effect was also shown to maintain activity against *P. aeruginosa* biofilms in combination with rifampicin, an antibiotic that normally cannot penetrate the outer membrane, and was effective against flat and mushroom-shaped biofilms (Armengol et al. 2023). In contrast, although this combination has demonstrated synergistic activity in *K. pneumoniae* biofilms, the enhanced susceptibility is insufficient to achieve colistin concentrations associated with clinical efficacy (Müderris et al. 2024). The lack of sustained bactericidal activity with triple combinations and levofloxacin–rifampicin in *S. aureus* biofilms may be explained by an antagonistic effect of rifampicin, due to its inhibition of RNA synthesis, which interferes with the action of quinolones on DNA (Murillo et al. 2008). Analysis of total CFU counts showed that bactericidal activity in mixed biofilms largely corresponded to the dominant species. The consistent failure to eradicate *S. aureus* across biofilm configurations underscores its central role in biofilm recalcitrance in this model. This may be related to the presence of persister cells, characterized by reduced metabolic activity, which are associated with the emergence of small-colony variants (SCVs) (Tran et al. 2023). In polymicrobial biofilms, other bacteria, such as *P. aeruginosa*, can promote SCV production in *S. aureus* (Trizna et al. 2020). The spatial distribution of bacteria further complicates treatment, as *S. aureus* remains localized in the deepest biofilm layers, limiting physical antibiotic penetration. In addition, polymicrobial interactions enhance recalcitrance through mechanisms such as horizontal gene transfer, β-lactamase-mediated antibiotic inactivation, quorum-sensing-driven transcriptional changes, inhibition of the electron transport chain, and alterations in membrane properties (Orazi and O’Toole 2019). These findings highlight the clinical relevance of accurately characterizing polymicrobial biofilms to guide the selection of effective antibiotic combination therapies.

The main limitation of this study is that all experiments were conducted using a single three-species biofilm model. All experiments used reference strains. Studies using clinical isolates are needed to validate the model, as these strains may better represent the microorganisms involved in PJI. Although this system was designed to mimic clinically relevant conditions, validation of the predictive equation and the fatty acid–based approach in additional polymicrobial models and in vivo settings is required to assess their broader applicability. Nevertheless, the originality of the proposed equation, the complementary value of fatty acid analysis, and the clinically relevant insights into antibiotic combination efficacy represent key strengths of this work.

## Conclusion

This study proposes a predictive model and evaluates the potential of a fatty acid–based approach for the characterization of polymicrobial biofilms. These methods complement spatial analysis and provide additional insight into how biofilm structure and species interactions may influence antimicrobial response. Integrative characterization may contribute to predicting treatment efficacy and to evaluating combination therapies in polymicrobial PJIs.

## Supplementary information

Below is the link to the electronic supplementary material.

Supplementary file 1 (PDF 4.65 MB)

## Data Availability

The data supporting the findings of this study are included within the article and its Online Resources. Additional raw data are available from the corresponding author upon reasonable request. Data have not been deposited in a public repository as all relevant datasets are either included in the manuscript or consist of large raw image files that are not suitable for standard repository deposition.

## Abbreviations

ATCC: American Type Culture Collection
BHI: Brain heart infusion
CAMHB: Cation-adjusted Müller–Hinton broth
CFU: Colony-forming unit
OD: Optical density
ODc: Optical density cutoff
PJIs: Prosthetic joint infections
PNA–FISH: Peptide nucleic acid fluorescence in situ hybridization
SCVs: Small-colony variants
TSA: Tryptic soy agar
TSB: Tryptic soy broth
