# Supplementary material for: Characterization and treatment of polymicrobial biofilms in prosthetic joint infections
Source: Appl Microbiol Biotechnol. 2026 Aug 4;110:329. doi: 10.1007/s00253-026-13985-w (PMC13724653; doi:10.1007/s00253-026-13985-w)
Supplement: Supplementary file 1 [file 253_2026_13985_MOESM1_ESM.pdf]

SUPPLEMENTARY MATERIAL

Journal: Applied Microbiology and Biotechnology

Title: Characterization and Treatment of Polymicrobial Biofilms in Prosthetic Joint Infections

Authors: Estíbaliz Torrecilla-Sádaba[a,1], Andreia S Azevedo[b], Daniel Carrizo[c], Isadora Maia[b], Laura Sánchez-García[c], Nuno F Azevedo[b], Jaime Esteban[a,d,f*], Llanos Salar-Vidal[a,d], John Jairo Aguilera-Correa[a,d,e]

Affiliations:

[a] Clinical Microbiology Department, IIS-Fundación Jiménez Díaz, Avenida de los Reyes Católicos, 2, 28040 Madrid, Spain

[b] Laboratory for Process Engineering, Environment, Biotechnology and Energy (LEPABE) and Associate Laboratory in Chemical Engineering (ALiCE), Faculty of Engineering, University of Porto, Rua Dr. Roberto Frias s/n, 4200-465 Porto, Portugal

[c] Centro de Astrobiología (CAB), CSIC-INTA, Calle Ajalvir, 28864 Torrejón de Ardoz, Madrid, Spain

[d] CIBERINFEC-CIBER de Enfermedades Infecciosas, Calle Monforte de Lemos, 3-5, Pabellón 11, Planta 0, 28029 Madrid, Spain

[e] Faculty of Biomedical and Health Sciences, Alfonso X el Sabio University (UAX), Avenida Universidad, 1, 28691 Villanueva de la Cañada, Madrid, Spain

[f] Department of Preventive Medicine, Public Health and Microbiology, Faculty of Medicine, Autonomous University of Madrid, Calle Arzobispo Morcillo, 4, Fuencarral-El Pardo, 28029 Madrid, Spain

Corresponding author:
Jaime Esteban
Department of Clinical Microbiology, Hospital Universitario Fundación Jiménez Díaz, Av. de los Reyes Católicos 2, 28040 Madrid, Spain
Email: jaime.esteban@uam.es

Table S1. Preparation of bacterial suspensions for mono-, dual-, and tri-species biofilms. Suspensions were prepared in sterile saline. Kp, *K. pneumoniae*; Pa, *P. aeruginosa*; Sa, *S. aureus*.

Biofilm	Bacteria	Volume (mL)*	Saline (mL)	Total Volume (mL)
Kp	Kp	1	1.44	2.44
	Pa	0		
	Sa	0		
Pa	Kp	0	1.94	2.44
	Pa	0.5		
	Sa	0		
Sa	Kp	0	1.5	2.44
	Pa	0		
	Sa	0.94		
Kp:Pa	Kp	1	0.94	2.44
	Pa	0.5		
	Sa	0		
Kp:Sa	Kp	1	0.5	2.44
	Pa	0		
	Sa	0.94		
Pa:Sa	Kp	0	1	2.44
	Pa	0.5		
	Sa	0.94		
Kp:Pa:Sa	Kp	1	0	2.44
	Pa	0.5		
	Sa	0.94		

*A 0.5 McFarland standard corresponded to: Kp ≃ 7.26 ± 0.01 CFU/mL; Pa ≃ 7.57 ± 0.17 CFU/mL; Sa ≃ 7.29 ± 0.06 CFU/mL.

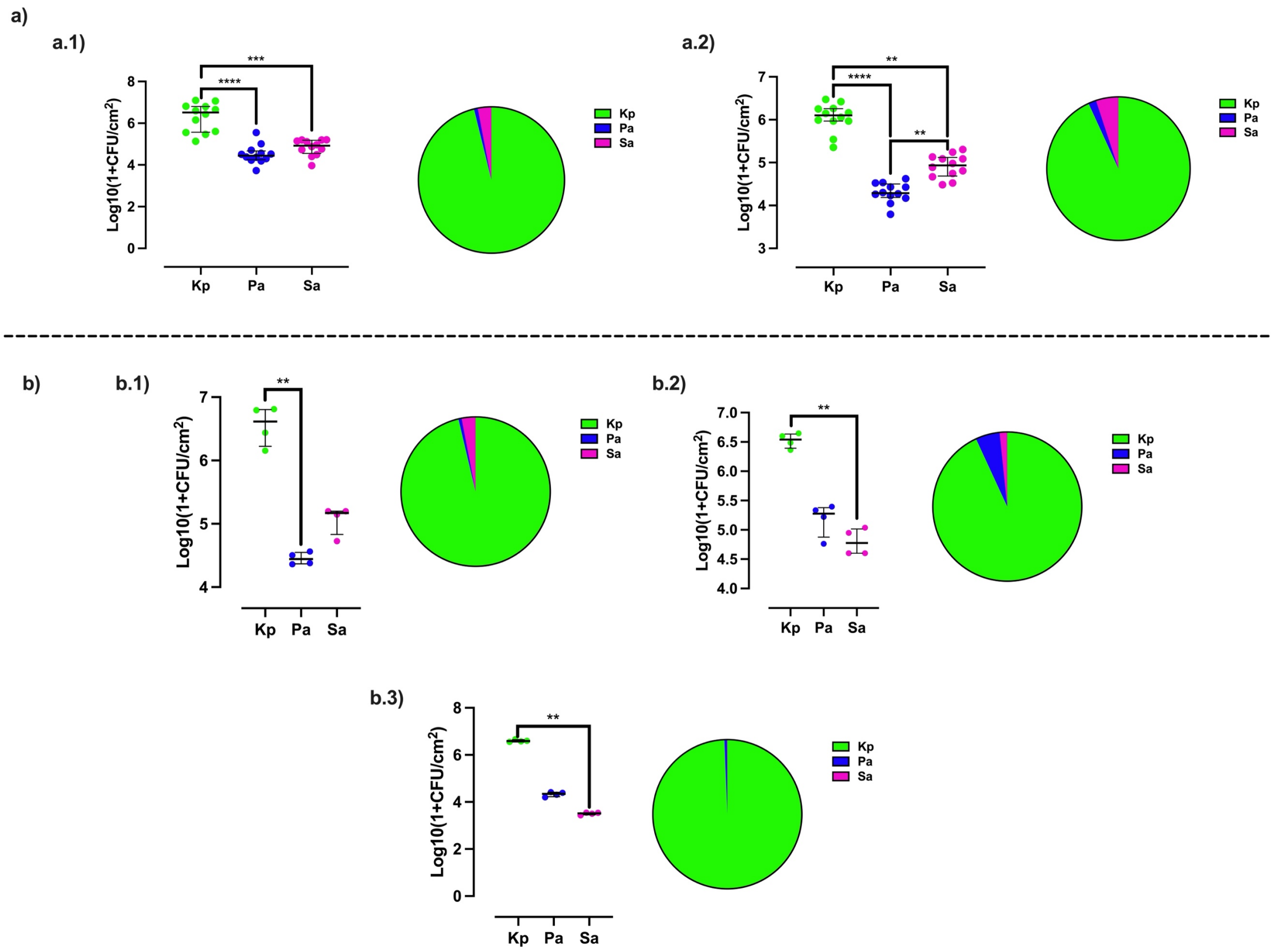

Fig. S1. Optimization of experimental conditions. (a) Broth medium: (a.1) brain–heart infusion (BHI) and (a.2) cation-adjusted Müller–Hinton broth (CAMHB). BHI was selected for subsequent experiments. (b) Bacterial inoculum concentrations in BHI: (b.1) 1 mL Kp, 0.5 mL Pa, and 0.94 mL Sa; (b.2) 0.05 mL Kp, 0.5 mL Pa, and 0.94 mL Sa; (b.3) 0.025 mL Kp, 0.5 mL Pa, and 0.94 mL Sa. Kp, *K. pneumoniae*; Pa, *P. aeruginosa*; Sa, *S. aureus*. Statistical significance is indicated as follows: *P < 0.05, **P < 0.01, ***P < 0.001, ****P < 0.0001. Data are presented as individual values with median and interquartile range. CFU, colony-forming units.

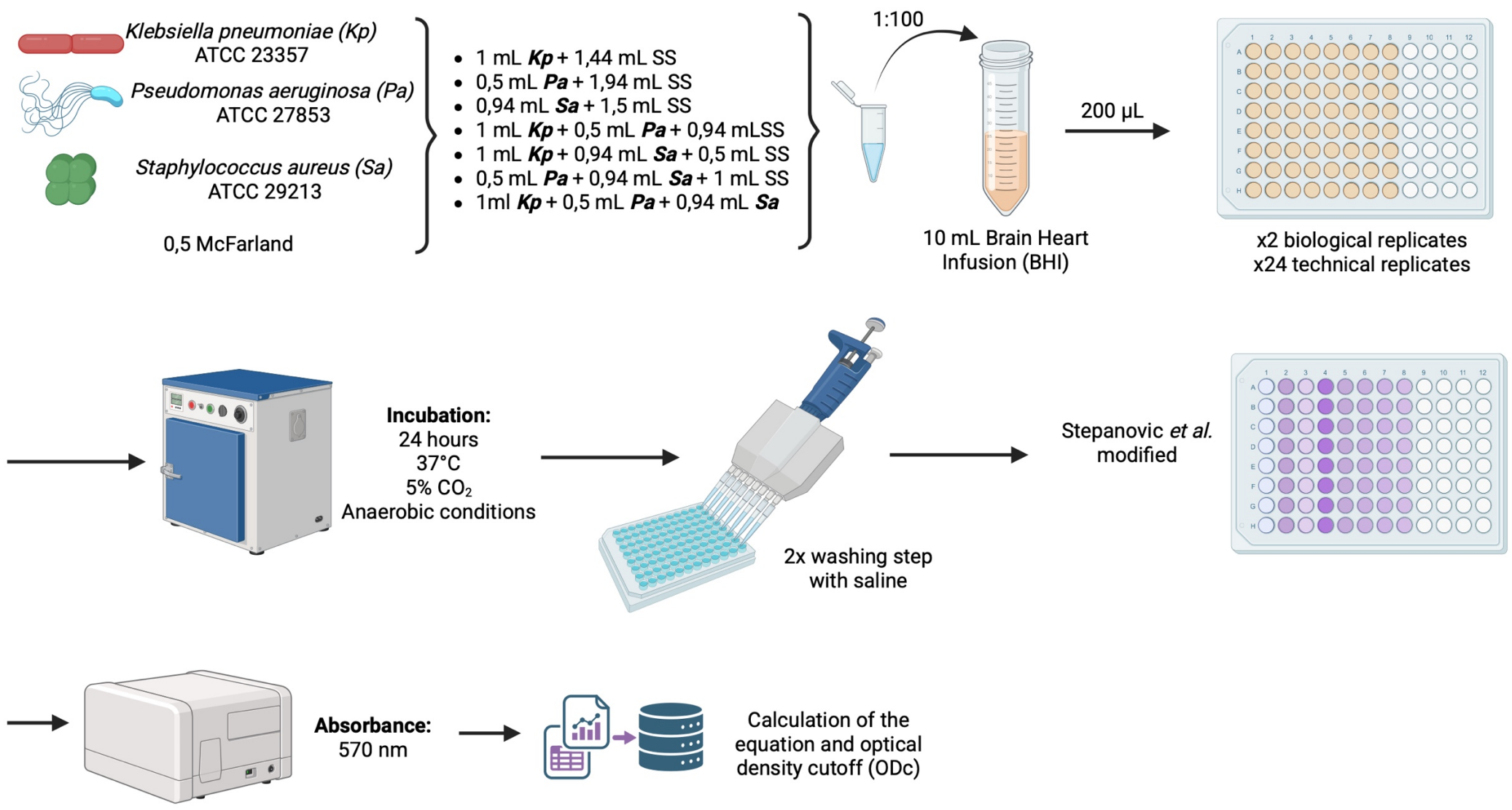

Fig. S2. Experimental workflow for predictive modelling of polymicrobial biofilm interactions. Schematic overview of biomass quantification and predictive equation development. Kp, *K. pneumoniae*; Pa, *P. aeruginosa*; Sa, *S. aureus*; SS, sterile saline. Created with BioRender.com.

ODc calculation (per microtiter plate)

$$ODc = mean\ OD\ of\ the\ negative\ control$$
$$+(3\ x\ SD\ of\ the\ negative\ control)$$

OD normalization of each strain

$$Final\ OD = men\ OD\ of\ each\ strain\ - ODc$$

Biofilm classification

$OD\ \leq ODc$	No biofilm producer
$ODc\ < OD\ \leq 2xODc$	Weak biofilm producer
$2xODc\ < OD\ \leq 4xODc$	Moderate biofilm producer
$OD\ > 4xODc$	Strong biofilm producer

Fig. S3. Calculation of the optical density (OD) and classification of biofilm production.

OD cutoff (ODc) was calculated for each microtiter plate using the negative control and applied to normalize OD values. Strains were subsequently classified into four categories according to OD thresholds.

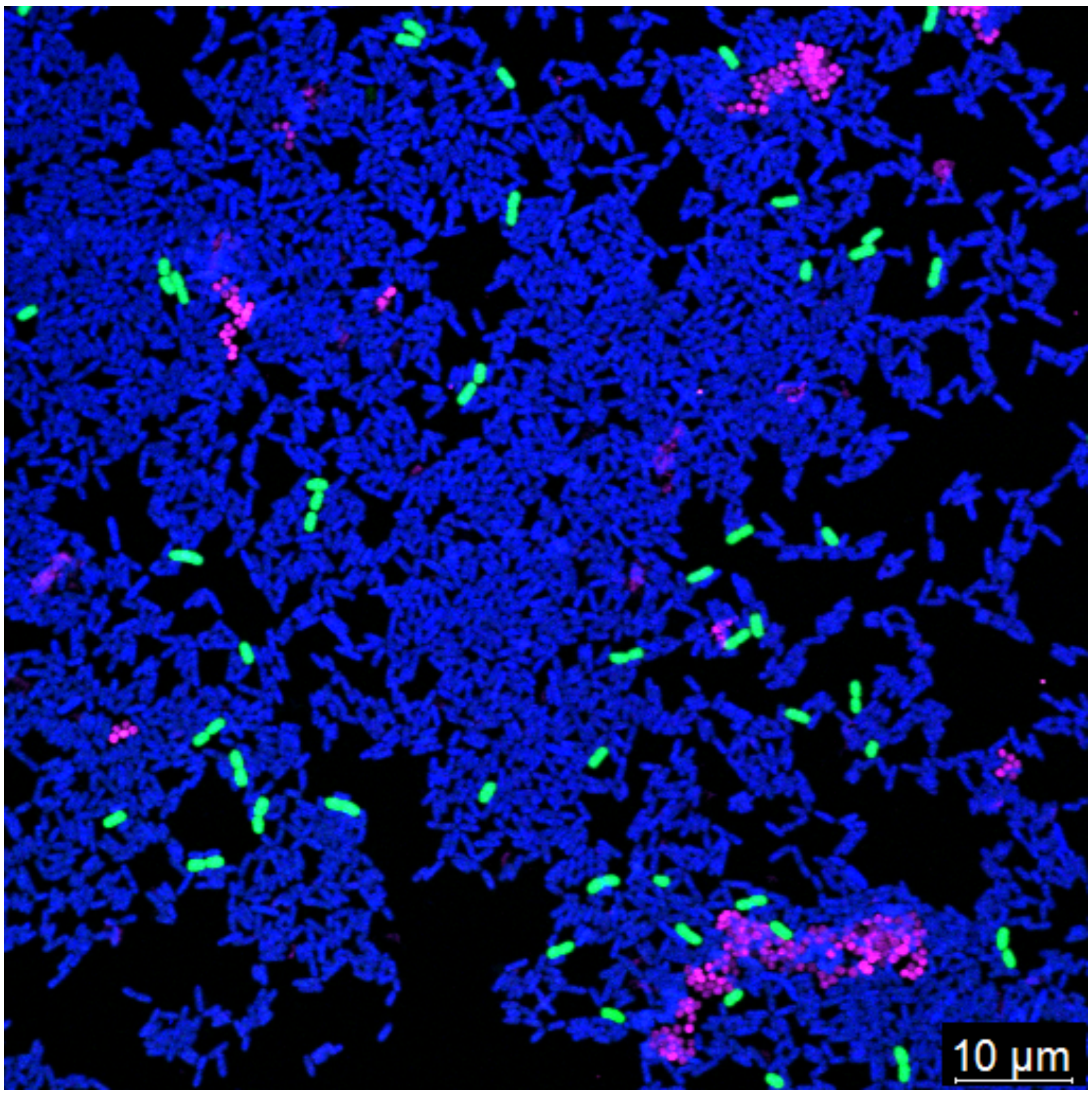

Fig. S4. Verification of PNA-FISH probe specificity and fluorescence channel separation. Mixed-cell suspension containing *K. pneumoniae* (green), *P. aeruginosa* (blue), and *S. aureus* (pink**).**

Table S2. Composition of buffers used in the PNA-FISH procedure.

The hybridization buffer was adjusted to pH 7.5 and sterilized by filtration (0.2 μm). The wash buffer was adjusted to pH 10 and sterilized by autoclaving.

**Buffer**	**Component**	**Concentration**
Hybridization buffer	Dextran sulfate	10% (w/v)
	NaCl	10 mM
	Formamide	30% (v/v)
	Sodium pyrophosphate	0.1% (w/v)
	Polyvinylpyrrolidone	0.2% (w/v)
	Ficoll	0.2% (w/v)
	EDTA di-sodium	5 mM
	Triton X-100	0.1% (v/v)
	Tris–HCl	50 mM
Wash buffer	Tris base	5 mM
	NaCl	15 mM
	Triton X-100	0.1% (v/v)

Table S3. Confocal microscopy acquisition parameters. Detailed imaging settings used for PNA-FISH experiments.

**Parameter**	**Specification**
Light source	Tunable white light laser (485–685 nm)
Internal detectors	Power HyD S (x2)
Objective	63× oil-immersion objective (63 ×/1.4 W)
Acquisition mode	XYZ scanning
Image resolution	1024 × 1024 pixels
Zoom factor	1 or 2
Pinhole size	1 Airy unit
Z-step interval	1 μm
Laser lines and emission detection ranges	491 nm (496–597 nm)
	551 nm (559–639 nm)
	626 nm (634–739 nm)

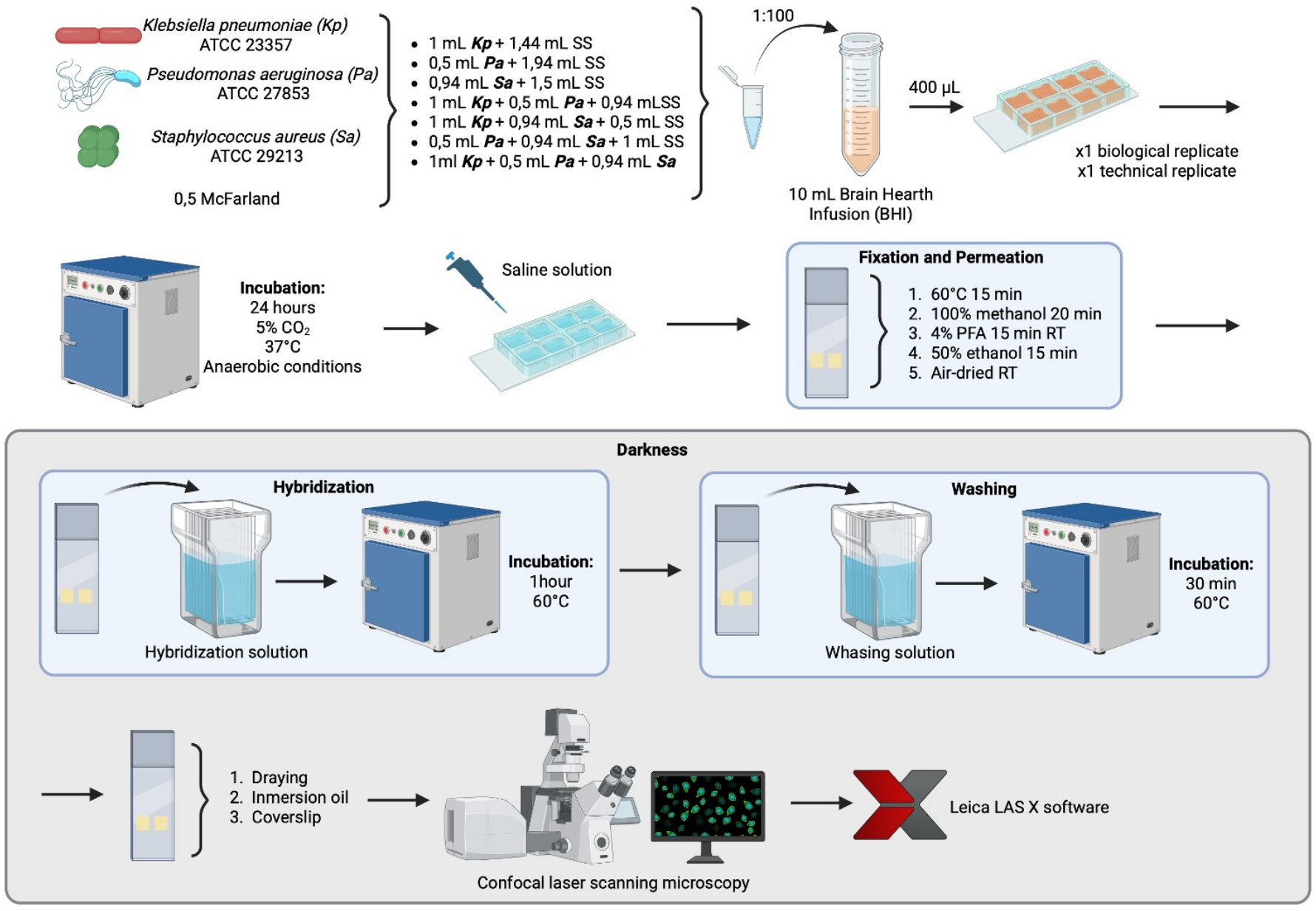

Fig. S5. Experimental workflow for spatial analysis of biofilms using PNA-FISH and confocal microscopy. Schematic overview of biofilm spatial analysis by PNA-FISH and confocal microscopy. Kp, *K. pneumoniae*; Pa, *P. aeruginosa*; Sa, *S. aureus*; SS, sterile saline; PFA, paraformaldehyde; RT, room temperature. Created with BioRender.com.

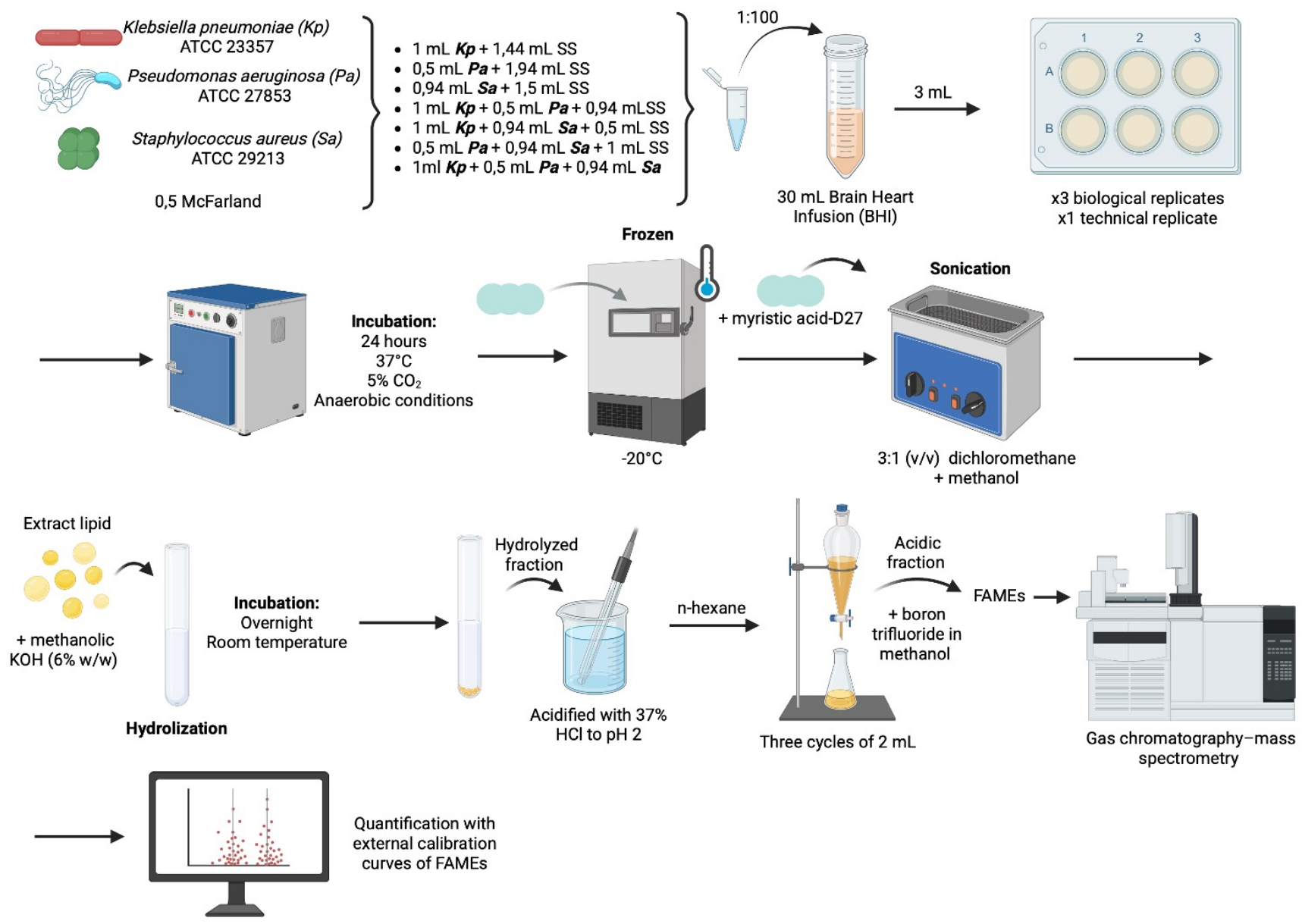

Fig. S6. Experimental workflow for fatty acid profiling of mono- and polymicrobial biofilms. Schematic overview of the experimental design used to assess the fatty acid composition of each biofilm. Kp, *K. pneumoniae*; Pa, *P. aeruginosa*; Sa, *S. aureus*; SS, sterile saline; FAMEs, fatty acid methyl esters. Created with BioRender.com.

Table S4. Detailed fatty acid extraction and GC–MS analytical conditions.

Specific reagents, conditions, and instrumental parameters not described in the main text are summarized. The GC oven temperature program was performed as previously described (Sánchez-García et al. 2018). GC-MS, gas chromatography–mass spectrometry; FAMEs, fatty acid methyl esters.

**Step**	**Parameter**	**Specification**
**Lipid extraction**	Solvent mixture	Dichloromethane:methanol (3:1, v/v)
	Internal standard	Myristic acid-D27
	Concentrated total lipid extract	Approximately 1 mL
**Hydrolysis**	Reagent	Methanolic KOH (6%, w/w)
	Conditions	Overnight, room temperature
**Acidification**	Reagent	HCl (37%)
	Final pH	2
**Extraction of acidic fraction**	Solvent	n-Hexane
	Cycles	3 × 2 mL
**Derivatization**	Reagent	Boron trifluoride in methanol
	Product	Fatty acid methyl esters (FAMEs)
**GC–MS system**	Instrument	Agilent 8860 GC + 5977B MSD
	Column	HP-5MS (30 m x 0.25 mm i.d. × 0.25 μm film thickness)
	Carrier gas	Helium
	Flow rate	1.1 mL·min^{-1}
**Identification**	Method	Reference standards (i.e., n-fatty acids)
		NIST library (MSD ChemStation software (v. 01.03.2357, Agilent Technologies))
**Quantification**	External calibration	Curves of FAMEs (C8–C24)

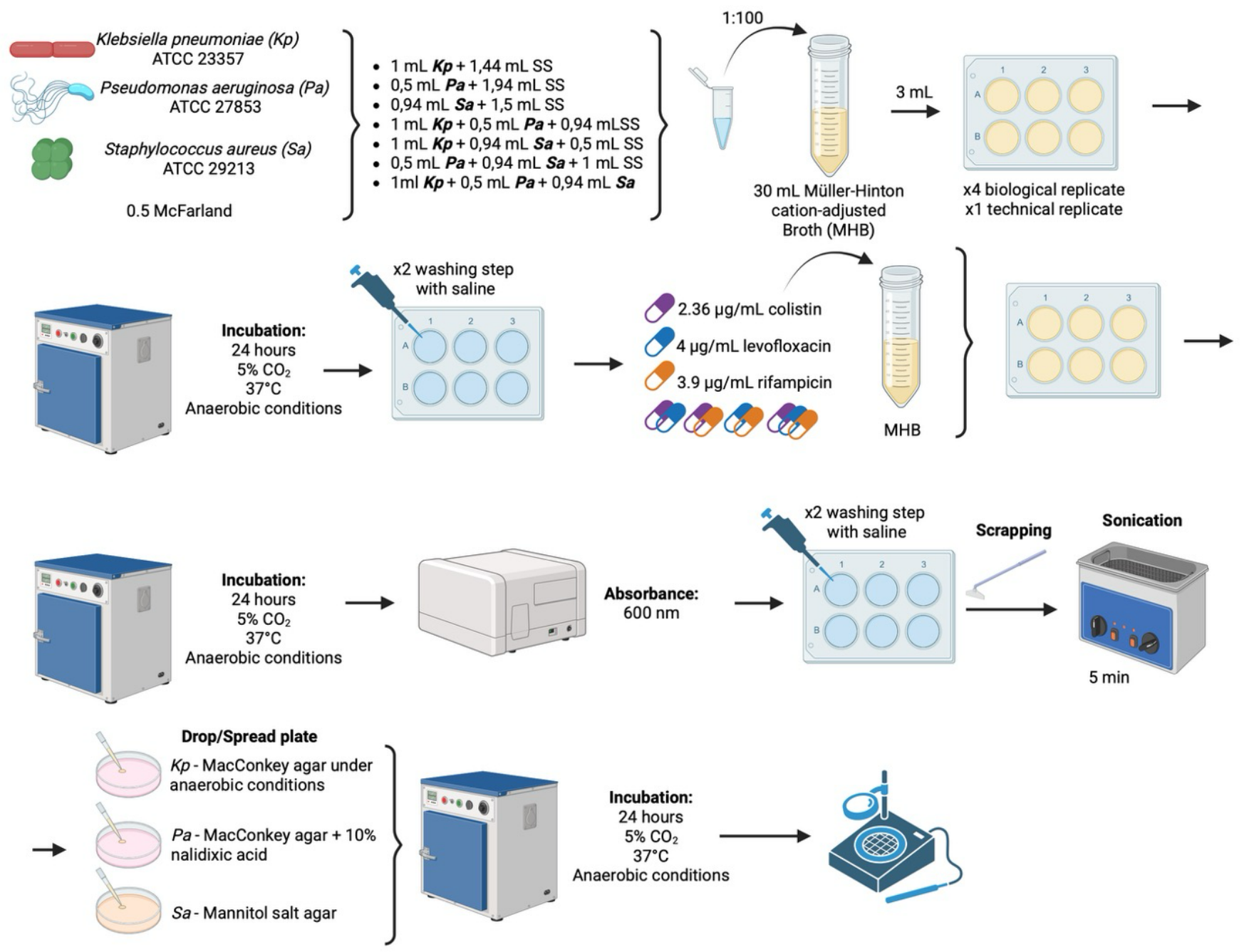

Fig. S7. Experimental workflow for antimicrobial treatment of mono- and polymicrobial biofilms. Schematic overview of the experimental design used for biofilm treatment. Kp, *K. pneumoniae*; Pa, *P. aeruginosa*; Sa, *S. aureus*; SS, sterile saline. Created with BioRender.com.

```
Dataset <- readXL("/Users/johnjairoaguileracorrea/Desktop/Stepanovic
1Kp+0,5Pa+0,94Sa 310523.xlsx",
+    rownames=FALSE, header=TRUE, na="", sheet="STATA",
stringsAsFactors=TRUE)

> Dataset <-
+   readXL("/Users/johnjairoaguileracorrea/Desktop/Stepanovic
1Kp+0,5Pa+0,94Sa 310523.xlsx",
+   rownames=FALSE, header=TRUE, na="", sheet="R",
stringsAsFactors=TRUE)

> LinearModel.2 <- lm(kppasa ~ kp + kppa + kpsa + pa + pasa + sa,
data=Dataset)

> summary(LinearModel.2)

Call:
lm(formula = kppasa ~ kp + kppa + kpsa + pa + pasa + sa, data =
Dataset)

Residuals:
    Min      1Q  Median      3Q     Max
-1.9888 -0.5564 -0.1219  0.3524  3.5161

Coefficients:
            Estimate Std. Error t value  Pr(>|t|)
(Intercept)  0.86986    1.92574   0.452    0.6539
kp           0.18628    0.33975   0.548    0.5865
kppa         0.68067    0.14984   4.543 0.0000482 ***
kpsa         0.58648    0.22428   2.615    0.0124 *
pa          -0.24219    0.62422  -0.388    0.7000
pasa         0.06155    0.03604   1.708    0.0953 .
sa          -0.17120    0.07416  -2.308    0.0261 *
---
Signif. codes:  0 '***' 0.001 '**' 0.01 '*' 0.05 '.' 0.1 ' ' 1

Residual standard error: 0.9822 on 41 degrees of freedom
Multiple R-squared:  0.7666,    Adjusted R-squared:  0.7325
F-statistic: 22.44 on 6 and 41 DF,  p-value: 1.625e-11


> LinearModel.3 <- lm(kppasa ~ kppa + kpsa + pasa + sa,
data=Dataset)

> summary(LinearModel.3)

Call:
lm(formula = kppasa ~ kppa + kpsa + pasa + sa, data = Dataset)

Residuals:
    Min      1Q  Median      3Q     Max
-1.9913 -0.5858 -0.0637  0.3246  3.5433

Coefficients:
```

```
            Estimate Std. Error t value  Pr(>|t|)
(Intercept)  1.22741    1.53523   0.799    0.4284
kppa         0.70513    0.14115   4.995 0.0000103 ***
kpsa         0.61525    0.21464   2.866    0.0064 **
pasa         0.06317    0.03462   1.825    0.0750 .
sa          -0.17778    0.07086  -2.509    0.0160 *
---
Signif. codes:  0 '***' 0.001 '**' 0.01 '*' 0.05 '.' 0.1 ' ' 1

Residual standard error: 0.9639 on 43 degrees of freedom
Multiple R-squared:  0.7643,    Adjusted R-squared:  0.7423
F-statistic: 34.85 on 4 and 43 DF,  p-value: 5.608e-13


> LinearModel.4 <- lm(kppasa ~ kppa + kpsa + sa, data=Dataset)

> summary(LinearModel.4)

Call:
lm(formula = kppasa ~ kppa + kpsa + sa, data = Dataset)

Residuals:
    Min      1Q  Median      3Q     Max
-2.2198 -0.6033 -0.2394  0.5269  3.0175

Coefficients:
            Estimate Std. Error t value    Pr(>|t|)
(Intercept)  1.35012    1.57383   0.858    0.395618
kppa         0.79211    0.13633   5.810 0.000000641 ***
kpsa         0.79017    0.19706   4.010    0.000232 ***
sa          -0.19900    0.07173  -2.774    0.008086 **
---
Signif. codes:  0 '***' 0.001 '**' 0.01 '*' 0.05 '.' 0.1 ' ' 1

Residual standard error: 0.989 on 44 degrees of freedom
Multiple R-squared:  0.746,     Adjusted R-squared:  0.7287
F-statistic: 43.08 on 3 and 44 DF,  p-value: 3.773e-13

Kp:Pa:Sa ~ 0.792 Kp:Pa + 0.79 Kp:Sa - 0.199 Sa
```

Fig. S8. Backward stepwise regression analysis for predictor selection based on AIC. R Commander console output of the backward stepwise regression used to identify predictors of multispecies biofilm formation according to the Akaike Information Criterion (AIC). Kp, *K. pneumoniae*; Pa, *P. aeruginosa*; Sa, *S. aureus*

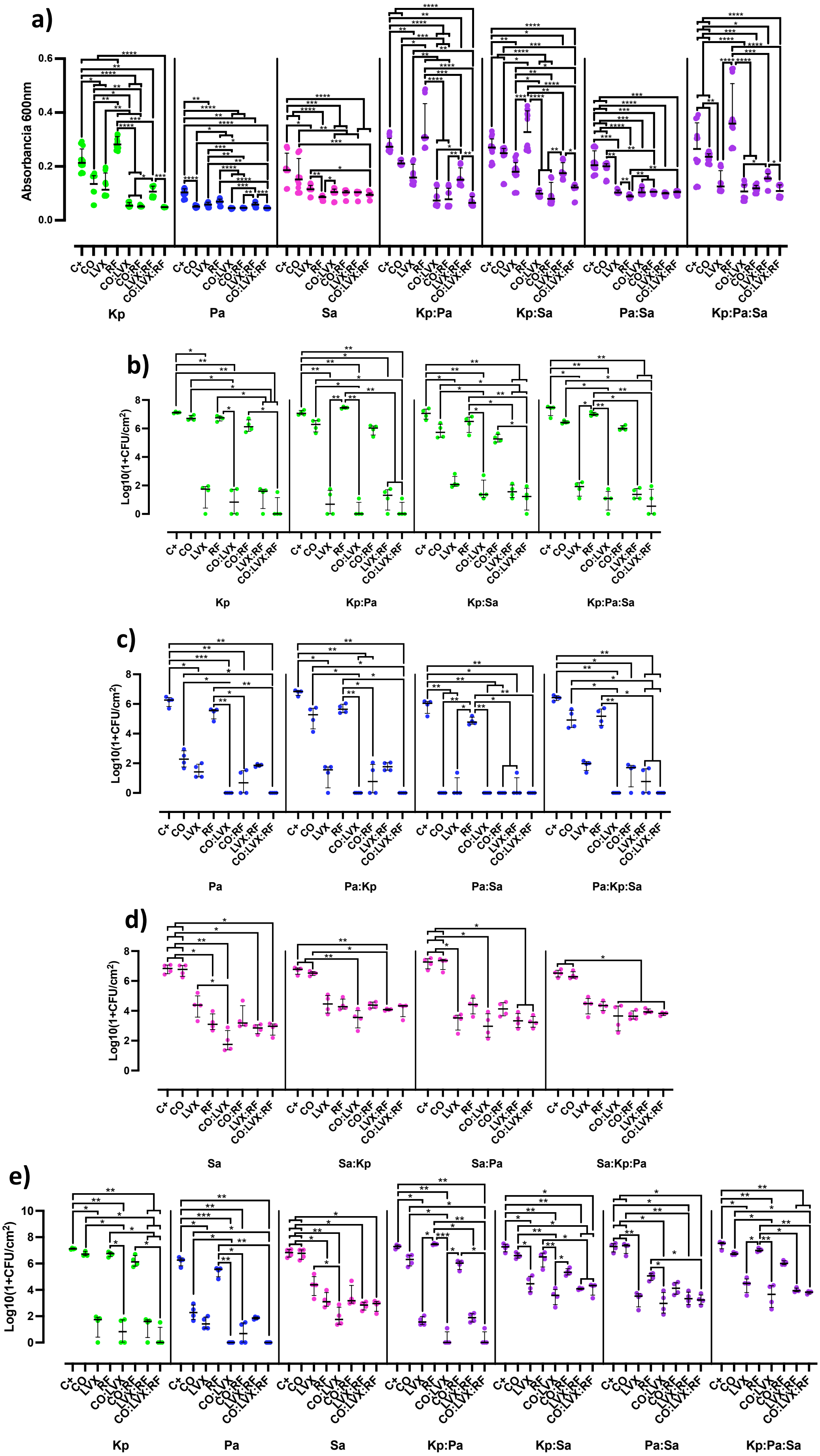
a)
Absorbancia 600nm
0.6
0.4
0.2
0.0
C+
CO
LVX
RF
CO:LVX
CO:RF
LVX:RF
CO:LVX:RF
Kp
Pa
Sa
Kp:Pa
Kp:Sa
Pa:Sa
Kp:Pa:Sa
b)
Log10(1+CFU/cm²)
8
6
4
2
0
Kp
Kp:Pa
Kp:Sa
Kp:Pa:Sa
c)
Log10(1+CFU/cm²)
Pa
Pa:Kp
Pa:Sa
Pa:Kp:Sa
d)
Log10(1+CFU/cm²)
Sa
Sa:Kp
Sa:Pa
Sa:Kp:Pa
e)
Log10(1+CFU/cm²)
10
Kp
Pa
Sa
Kp:Pa
Kp:Sa
Pa:Sa
Kp:Pa:Sa

Fig. S9. Statistically significant differences among antimicrobial treatments. This figure displays all statistically significant pairwise comparisons among treatments, whereas the main text reports significance relative to the control condition only. (a) Optical density at 600 nm of planktonic cells. (b) CFU/cm² of *K. pneumoniae* (Kp). (c) CFU/cm² of *P. aeruginosa* (Pa). (d) CFU/cm² of *S. aureus* (Sa). (e) Total CFU/cm² (sum of all species). C+, positive control; CO, colistin; LVX, levofloxacin; RF, rifampicin. Statistical significance is indicated as follows: *P < 0.05, **P < 0.01, ***P < 0.001, ****P < 0.0001. Data are presented as individual values with median and interquartile range.

REFERENCES

Sánchez-García L, Aeppli C, Parro V, Fernández-Remolar D, García-Villadangos M, Chong-Diaz G, Blanco Y, Carrizo D (2018) Molecular biomarkers in the subsurface of the Salar Grande (Atacama, Chile) evaporitic deposits. Biogeochemistry 140:31–52. https://doi.org/10.1007/s10533-018-0477-3
